# Blood prognostic predictors of treatment response for patients with papillary thyroid cancer

**DOI:** 10.1042/BSR20202544

**Published:** 2020-10-21

**Authors:** Xiangxiang Liu, Zhongke Huang, Xianghui He, Xiangqian Zheng, Qiang Jia, Jian Tan, Yaguang Fan, Cen Lou, Zhaowei Meng

**Affiliations:** 1 Department of Nuclear Medicine, Tianjin Medical University General Hospital, Tianjin, P.R. China; 2 Department of Nuclear Medicine, Sir Run Run Shaw Hospital Affiliated to School of Medicine, Zhejiang Universtity, Hangzhou, Zhejiang, P.R. China; 3 Department of General Surgery, Thyroid Surgery Division, Tianjin Medical University General Hospital, Tianjin, P.R. China; 4 Department of Thyroid and Neck Tumor, Tianjin Medical University Cancer Institute and Hospital, National Clinical Research Center for Cancer, Key Laboratory of Cancer Prevention and Therapy, Tianjin, P.R. China; 5 Tianjin Key Laboratory of Lung Cancer Metastasis and Tumor Microenvironment, Tianjin Lung Cancer Institute, Tianjin Medical University General Hospital, Tianjin, P.R. China

**Keywords:** Papillary thyroid cancer (PTC), Prognosis, Platelet (PLT)

## Abstract

Background: Papillary thyroid cancer (PTC) is a very common malignant disease with high morbidity. We needed some pretreatment indicators to help us predict prognosis and guide treatment. We conducted a study about some pretreatment prognostic indicators.

Methods: This clinical study recruited 705 postoperative PTC patients (211 males, 494 females). Clinical data before radioactive iodine (RAI) treatment were collected. Patients’ response to therapy were classified into two categories: ‘Good Prognosis Group’ (GPG) and ‘Poor Prognosis Group’ (PPG), according to ‘2015 American Thyroid Association Guidelines’. Differences of indicators between different prognosis groups were compared. Odds ratios (ORs) were calculated by univariate/multiple binary logistic regression models. Difference of body mass index (BMI) changes before and after RAI treatment between different prognosis groups was also compared.

Results: A total of 546 (77.45%) belonged to GPG, and 159 (22.55%) belonged to PPG. Platelet (PLT), neutrophil (NEUT), PLT subgroups, and combination of red blood cell distribution width (RDW) and BMI (COR-BMI) were different between two prognosis groups. The significance of the difference between the two groups of BMI disappeared after the Bonferroni correction. PLT and PLT subgroups had detrimental effects on the risk of PPG; T stage had a positive effect on the risk of PPG. PLT subgroup showed a detrimental effect on the risk of PPG when we included additional covariates.

Conclusions: We found that lower pretreatment PLT levels may indicate a poor prognosis for PTC. The relationship between platelet-derived growth factor (PDGF) and radiation sensitivity may be the key to this association.

## Introduction

Thyroid cancer is the most common endocrine system malignant disease, accounting for 90% of all endocrine cancer cases [1,2]. In the past few decades, the incidence of thyroid cancer has risen rapidly in many countries in the world, including China [3,4]. The incidence in Korea has increased the most: the incidence among people 15–79 years of age (standardized to the world population) increased from 12.2 per 100000 in 1993–1997 to 59.9 per 100000 in 2003–2007 [1]. This rapid growth has caused widespread public concern about thyroid cancer. Thyroid cancer can be classified according to its histopathological characteristics, mainly including papillary thyroid cancer (PTC), follicular thyroid cancer, medullary thyroid cancer, and anaplastic thyroid cancer (ATC) [5]. Among all pathological types of thyroid cancer, PTC is the most common and least invasive type of histology, contributing to the highest morbidity increase [1,3,4,6]. For most patients, total thyroidectomy, ablation of tumor remnants by radioactive iodine (RAI) therapy and thyroid stimulating hormone (TSH) suppression therapy are the main steps in the treatment of PTC [7]. After this standardized treatment process, patients presented different outcomes: some patients were cured or stable; some patients’ disease progressed, and some patients were even distant metastasized. For the difference in patient prognosis, some indicators that were predictive of prognosis needed to be proposed to help us predict prognosis and guide treatment [8,9]. The goal of this strategy was to detect recurrent disease early, identify patients who would benefit from further treatment, and reduce over-investigation of low-risk patients.

Several factors can predict the prognosis of PTC patients [10,11]. In clinical practice, the most commonly used prognostic predictor of PTC patients is the 2015 American Thyroid Association (ATA) staging system, but its predictive power is still far from perfect [12,13]. The initial risk stratification system may not be able to accurately predict persistent diseases and recurrence during the follow-up. The prognosis of cancer patients is closely related to the response to treatment. Some gene mutations, such as proto-oncogene B-Raf (BRAF) and telomerase reverse transcriptase (TERT), have been found in more invasive subsets of PTC [14,15]. However, these gene mutations are rarely used in routine clinical practice because of their high cost and limited availability. Therefore, it is necessary to determine effective and reliable clinical prognostic parameters.

Chronic inflammation usually occurs in malnourished patients and is associated with poor prognosis [16,17]. Nutritional index and inflammatory markers or inflammation-based prognostic scores may be reliable, practical prognostic tools for a variety of malignancies, including PTC. Studies have shown that higher red blood cell distribution width (RDW) and lower body mass index (BMI) are markers of malnutrition and chronic inflammation in cancer patients [18,19]. Combination of RDW and BMI (COR-BMI) has been shown to be an important independent prognostic factor affecting tumor-specific survival in patients with laryngeal squamous cell carcinoma and nasopharyngeal carcinoma (NPC) [18,19]. Recent publications have suggested that platelet (PLT) count is related to prognosis in many cancers [20,21]. In addition, some inflammatory markers, such as neutrophil–lymphocyte ratio (NLR), macrophages, and tumor-infiltrating mast cells, have been reported to be associated with a poor prognosis in PTC [22–24].

However, the ability of some predictions in PTC has not been well studied. Therefore, the purpose of the present study was to investigate the ability of these indicators to predict prognosis in patients with PTC.

## Materials and methods

### Design

**Figure 1:**
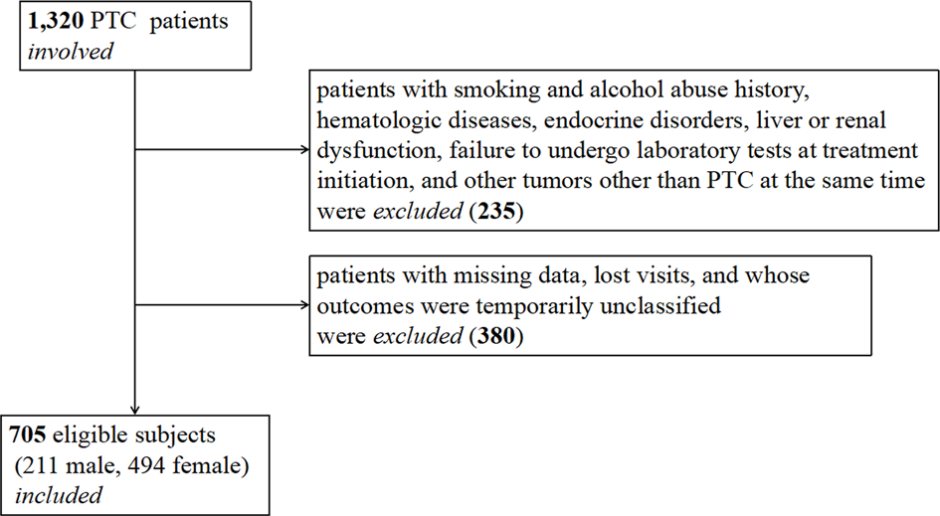
Patient screening process

This clinical study was conducted at the Department of Nuclear Medicine, General Hospital of Tianjin Medical University in China. From August 2008 to August 2018, a total of 1320 PTC postoperative patients, who were scheduled to receive RAI therapy in the nuclear medicine ward, participated in the present study; refer to our previous researches [25–30]. All participants were asked to complete a questionnaire about medical history, lifestyle, alcohol intake, and smoking. To avoid the influence of confounding factors, the exclusion criteria were smoking and alcohol abuse history, hematologic diseases, endocrine disorders, liver or renal dysfunction, failure to undergo laboratory tests before RAI therapy, and tumors other than PTC at the same time. In addition, patients with missing data, lost visits, and whose outcomes were temporarily unclassified were excluded. Finally, 705 eligible subjects (211 males, 494 females) were included. The screening process of the patients is shown in Figure 1.

### Measurements

Demographics, anthropometric measurements, and peripheral blood indicators were collected from all subjects on the first day of their hospitalization, before RAI therapy. Fasting blood samples were obtained between 7 and 10 a.m. Height and weight were measured in centimeters and kilograms, respectively. BMI was calculated by dividing weight (kg) by height squared (m^2^). TSH and thyroglobulin (Tg) were analyzed on a fully automated ADVIA Centaur Analyzer (Siemens Healthcare Diagnostics, Erlangen, Germany) based on a chemiluminescent reaction principle. Hemoglobin (Hg), Neutrophil (NEUT), lymphocyte (LBC), PLT, plateletcrit (PCT), mean platelet volume (MPV), platelet distribution width (PDW), RDW, and albumin (ALB) were determined enzymatically by an auto-analyzer (Hitachi Model 7600 analyzer, Hitachi, Tokyo, Japan). These variables were selected for potential relationship to the prognosis of PTC based on previous studies. Follow-up data on patient BMI changes were collected based on patient outpatient review.

Surgical specimens were microscopically examined by two or more experienced pathologists who assessed the following histopathological factors: the histological type of the primary lesion, the size of the primary tumor (measuring the longest diameter of the largest lesion), location, multifocal, extrathyroidal extension, lymphatic invasion, marginal involvement, lymph node metastasis, and potential thyroid disease such as chronic lymphocytic thyroiditis. The pathological variants other than conventional PTC included: follicular variant of PTC, oncocytic variant of PTC, diffuse sclerosing variant of PTC, tall cell variant of PTC, columnar cell variant of PTC, and solid variant of PTC. Post-treatment/Diagnostic whole-body RAI scans were performed by SPECT/CT (Discovery NM/CT 670, GE Healthcare, Chicago, America).

### Definitions

For the RDW, a cutoff of 13.25 was generated according to the receiver operating characteristic (ROC) analysis in the training set for CSS (sensitivity 71.8%, specificity 33.3%, area under the curve [AUC] 0.554, 95% confidence interval [CI] 0.504–0.64, *P*<0.001). RDW values were categorized into two groups: RDW ≤ 13.25 and RDW *>* 13.25 (%). Similarly, NLR values were categorized into two groups: NLR ≤ 2.23 and NLR *>* 2.23. PLT values were categorized into two groups: PLT ≤ 302 and PLT *>* 302 (×10^9^/l). It has been suggested that the BMI cut-off point for overweight or obesity in the Chinese population should be lower than WHO standards. Therefore, we adopted the Chinese BMI cut-off values proposed by the Working Group on Obesity in China described in the Guidelines for Prevention and Control of Overweight and Obesity in Chinese Adults to define overweight or obesity [31,32]. In our study, BMI was categorized into three groups: BMI *≤* 18.5, 18.5 < BMI *≤* 24, and BMI > 24 (kg/m^2^). For the COR-BMI, patients with RDW > 13.25 and BMI *≤* 18.5 were defined as COR-BMI 1. Patients with RDW ≤ 13.25 and BMI *≤* 18.5 or 18.5 < BMI *≤* 24, and patients with RDW *>* 13.25 and 18.5 < BMI *≤* 24 or BMI > 24 were defined as COR-BMI 2. Patients with RDW ≤ 13.25 and BMI > 24 were defined as COR-BMI 3. According to ‘2015 ATA Management Guidelines for Adult Patients with Thyroid Nodules and Differentiated Thyroid Cancer’ [7], patients’ response to therapy can be classified into two categories: Excellent response and Indeterminate response were defined as ‘Good Prognosis Group’ (GPG). Biochemical incomplete response and Structural incomplete response were defined as ‘Poor Prognosis Group’ (PPG) (Table 1). BMI changes were compared between the first and last times they were hospitalized.

**Table 1:** Clinical implications of response to therapy reclassification in patients with differentiated thyroid cancer treated with total thyroidectomy and radioiodine remnant ablation

Binary variable	Category	Definitions	Clinical outcomes
GPG	Excellent response	Negative imaging and either suppressed Tg < 0.2 ng/ml^1^ or TSH-stimulated Tg < 1 ng/ml^1^	1–4% recurrence <1% disease-specific death
	Indeterminate response	Non-specific findings on imaging studies Faint uptake in thyroid bed on RAI scanning Non-stimulated Tg detectable, but <1 ng/ml Stimulated Tg detectable, but <10 ng/ml or Anti-Tg antibodies stable or declining in the absence of structural or functional disease	15%–20% will have structural disease identified during follow-up In the remainder, the non-specific changes are either stable, or resolve <1% disease-specific death
PPG	Biochemical incomplete response	Negative imaging And Suppressed Tg ≥ 1 ng/ml^1^ Or Stimulated Tg ≥ 10 ng/ml^1^ Or Rising anti-Tg antibody levels	At least 30% spontaneously evolve to NED 20% achieve NED after additional therapy 20% develop structural disease <1% disease-specific death
	Structural incomplete response	Structural or functional evidence of disease With any Tg level With or without anti-Tg antibodies	50–85% continue to have persistent disease despite additional therapy Disease-specific death rates as high as 11% with loco-regional metastases and 50% with structural distant metastases

1 In the absence of anti-Tg antibodies.

Abbreviation: NED, a patient having no evidence of disease at final follow-up.

### Statistical analysis

Continuous variables with normal distribution were presented as mean with standard deviation (SD), and the *t* test was used to compare between-group difference. Continuous variables with skewed distribution were presented as median with interquartile range (IQR), and Mann–Whitney U test was used to compare between-group difference. Categorical variables were presented as frequencies with percentages, and Chi-square test and Fisher’s exact test was used to compare between-group difference. For the pairwise comparison between groups greater than two, we adjusted the significance level according to Bonferroni’s method to reduce the risk of type I errors. After stratifying data by COR-BMI, NLR subgroups and PLT subgroups, odds ratios (ORs) for PPG with 95% CIs were calculated by univariate binary logistic regression. Further, multiple binary logistic regression calculated ORs for PPG with 95% CIs after adding other covariates. Difference of BMI changes before and after RAI treatment between different prognosis groups was also compared by Mann–Whitney U test. The analyses were performed with Statistical Package for Social Sciences (SPSS version 22.0; SPSS Inc., Chicago, IL). Differences were considered significant at *P*<0.05.

## Results

### Baseline characters between patients in different prognosis groups

The clinical characteristics of participants were summarized in Table 2. Those 705 eligible subjects included 211 males and 494 females. Among them, 546 (77.45%) belonged to GPG, and 159 (22.55%) belonged to PPG. PLT was higher in GPG than PPG (265.36 ± 63.62 vs. 251.20 ± 63.25, *P*<0.05). NEUT was lower in GPG than PPG (3.85 [2.95–3.85] vs. 4.47 [3.16–5.83], *P*<0.05). Lower PLT subgroups had a higher prevalence of PPG (24.4% vs. 18.0%, *P*<0.05). The distribution of COR-BMI between two prognosis groups was different (*P*<0.05). We further compared the differences between each two groups using the Bonferroni’s method. The significance level was adjusted to *P*<0.017. We found no significant differences between each of the two groups (differences between COR-BMI 1 and 2, 1 and 3, and 2 and 3: *P*>0.017). There was no significant difference in other variables between two prognosis groups (*P*>0.05).

**Table 2:** Comparison of clinical characteristics of patients with different prognosis groups

Characteristics	Total	Good curative effect	Poor curative effect	*P*-value
Number	705	546 (77.45%)	159 (22.55%)	
**Continuous variables with normal distribution**				
Age	45.02 ± 11.46	44.87 ± 10.86	45.53 ± 13.36	0.573
Hg	139.61 ± 16.04	139.44 ± 16.36	140.19 ± 14.92	0.609
ALB	46.20 ± 3.17	46.26 ± 3.27	45.97 ± 2.77	0.279
PLT	261.95 ± 63.81	265.36 ± 63.62	251.20 ± 63.25	0.014^1^
PDW	12.32 ± 1.65	12.20 ± 2.12	12.48 ± 2.06	0.135
MPV	10.49 ± 0.75	10.47 ± 0.92	10.58 ± 0.91	0.177
RDW	13.37 ± 2.73	13.59 ± 5.72	13.11 ± 1.26	0.293
BMI	25.44 ± 3.19	25.45 ± 5.04	25.42 ± 4.21	0.945
**Continuous variables with skewed distribution**				
NEUT	3.99 (3.00–5.23)	3.85 (2.95–3.85)	4.47 (3.16–5.83)	0.008^1^
LBC	2.02 (1.57–2.92)	1.95 (1.54–2.65)	2.06 (1.51–6.15)	0.119
PCT	0.28 (0.24–0.33)	0.28 (0.24–0.33)	0.29 (0.23–0.33)	0.159
NLR	2.11 (1.56–2.31)	2.11 (1.54–3.32)	2.11 (1.71–2.19)	0.448
**Categorical variables**				
Gender				0.781
Male	211	162 (76.8%)	49 (23.2%)	
Female	494	384 (77.7%)	110 (22.3%)	
Variants				0.674
Yes	48	36 (75.0%)	12 (25.0%)	
No	657	510 (77.6%)	147 (22.4%)	
PLT subgroups				0.001^1^
1	550	411 (74.7%)	139 (25.3%)	
2	155	135 (87.1%)	20 (12.9%)	
NLR subgroups				0.069
1	505	382 (75.6%)	123 (24.4%)	
2	200	164 (82.0%)	36 (18.0%)	
T stage				0.110^2^
1a	207	170 (82.1%)	37 (17.9%)	
1b	273	210 (76.9%)	63 (23.1%)	
2	51	42 (82.4%)	9 (17.6%)	
3	99	71 (71.7%)	28 (28.3%)	
4a	58	43 (74.1%)	15 (25.9%)	
4b	17	10 (58.8%)	7 (41.2%)	
N stage				0.168
0	99	82 (82.8%)	17 (17.2%)	
1a	369	289 (78.3%)	80 (21.7%)	
1b	237	175 (73.8%)	62 (26.2%)	
COR-BMI				0.047^1^^,^^2^
1	9	6 (66.7%)	3 (33.3%)	0.386^2^^,^^3^
2	371	300 (80.9%)	71 (19.1%)	0.703^3^^,^^4^
3	325	240 (73.8%)	85 (26.2%)	0.027^5^

^3,4,5^Adjusted the significance level according to Bonferroni’s method. *P*<0.017 is considered significant.

1 *P*<0.05.

2 Fisher’s exact test.

3 Differences between COR-BMI 1 and 2.

4 Differences between COR-BMI 1 and 3.

5 Differences between COR-BMI 2 and 3.

### Risks of PGP

We used binary logistic regression to model the risks of PPG (Table 3). PLT had a detrimental effect on the risk of PPG (OR = 0.996, 95% CI = 0.993–0.999, *P*<0.05). T stage had a positive effect on the risk of PPG (OR = 1.239, 95% CI = 1.084–1.417, *P*<0.05). PLT subgroups had a detrimental effect on the risk of PPG (OR = 0.438, 95% CI = 0.264–0.728, *P*<0.05). Among the other variables, there was no significant difference in the risk of PPG (*P*>0.05).

**Table 3:** Risk of PGP with different variables

Variables	OR (95% CI)	*P*-value
Age	1.005 (0.990–1.021)	0.526
Hg	1.003 (0.992-1.014)	0.609
PLT	0.996 (0.993–0.999)	0.009^1^
ALB	0.973 (0.323–0.973)	0.323
NEUT	0.988 (0.966–1.012)	0.331
LBC	1.008 (0.985–1.032)	0.483
NLR	1.097 (0.951–1.267)	0.204
Gender	1.056 (0.720–1.549)	0.781
T stage	1.239 (1.084–1.417)	0.002^1^
N stage	1.299 (0.990–1.704)	0.060
Variants	1.156 (0.587–1.156)	0.675
NLR subgroups	0.682 (0.451–1.031)	0.070
PLT subgroups	0.438 (0.264–0.728)	0.001^1^
COR-BMI	1.380 (0.981–1.941)	0.065

1 *P*<0.05.

Furthermore, we calculated risks for PPG with multiple binary logistic regressions (Table 4). Different models were stratified by COR-BMI, NLR subgroups, and PLT subgroups. Table 4 shows the confounding factors for each model. We found that COR-BMI 2 subgroups had a detrimental effect on the risk of PPG when COR-BMI 3 subgroups were used as reference (*P*<0.05). This effect still existed when we included additional covariates (*P*<0.05). Higher PLT subgroups showed a detrimental effect on the risk of PPG compared with the lower subgroup (*P*<0.05), when confounding factors were added or no confounding factors were added. While NLR subgroups showed no significant differences on the risk of PPG in both models (*P*>0.05).

**Table 4:** Risk of PGP

Variables	Crude OR		Adjusted OR	
COR-BMI^2^	OR (95% CI)	*P*-value	OR (95% CI)	*P*-value
1	1.412 (0.345–5.770)	0.631	1.326 (0.319–5.517)	0.698
2	0.668 (0.467–0.956)	0.027^1^	0.632 (0.437–0.915)	0.015^1^
3	Reference		Reference	
NLR subgroups^3^				
NLR ≤ 2.23	Reference		Reference	
NLR*>* 2.23	0.682 (0.451–1.031)	0.070	0.698 (0.455–1.070)	0.099
PLT subgroups^4^	(×10^9^/l)			
PLT ≤ 302	Reference		Reference	
PLT *>* 302	0.438 (0.264–0.728)	0.001^1^	0.426 (0.254–0.714)	0.001^1^

Crude ORs were calculated by univariate binary logistic regressions; adjusted ORs were calculated by multiple binary logistic regressions.

1 *P*<0.05.

2 Confounding factors in the multiple binary logistic regression included PLT, T stage, N stage, and NLR subgroups.

3 Confounding factors in the multiple binary logistic regression included COR-BMI, PLT, T stage, and N stage.

4 Confounding factors in the multiple binary logistic regression included COR-BMI, T stage, N stage, and NLR subgroups.

### BMI changes before and after treatment between patients in different prognosis group

Overall, BMI changes in all eligible subjects ranged from −8.8 to 6.25, with mean and SD of −0.50 ± 2.32. Difference between two BMI changes curative effect groups was not significant (−0.55 ± 2.17 vs. −0.48 ± 2.39, *P*>0.05). Furthermore, we used binary logistic regression to calculate the risk of PPG. The result was not significant (OR = 1.014, 95% CI = 0.802–1.282, *P*>0.05).

## Discussion

A lot of research on the predictive effect of indicators on the prognosis of malignant tumors has been done in other tumors. For example, the prognostic nutritional index have been reported to predict prognosis in NPC [33] and hepatocellular carcinoma [17]; NLR have been reported in NPC [34] and colorectal cancers [35]; mast cells have been reported in prostate cancer [36]; serum C-reactive protein, platelet–lymphocyte ratio, and lymphocyte–monocyte ratio were important predictors of prognosis of NPC [33,34]. High RDW presents a prognostic value of lower survival in oncological patients with gastric, lung, renal, and hematological neoplasia [37–39]. However, prognostic indicators of PTC are understudied. To the best of our knowledge, this is the first study to investigate the predictive role of COR-BMI and PLT in PTC patients. It is also the first study to evaluate PLT as an independent prognostic indicator for PTC patients.

As we all know, PTC has a high morbidity but most patients have a good prognosis [1,6]. GPG has a good prognosis and a high propensity to reach long-term remission [7]. In our study, GPG accounted for 77.45% and PPG accounted for 22.55%, similar to other studies [2,7]. We found PLT, NEUT, and COR-BMI were different in two prognosis groups (*P*<0.05). However, we adjusted the significance levels of COR-BMI according to the Bonferroni’s method and compared the two groups. We did not find significant differences between the two groups (*P*>0.017). Even though we found COR-BMI 2 subgroups had a detrimental effect on the risk of PPG when COR-BMI 3 was used as reference (*P*<0.05). This may be an error that is not corrected by statistics. Therefore, we cannot conclude that there is a correlation between COR-BMI and PTC prognosis based on the above results.

COR-BMI is a new indicator of the combination of inflammation and nutritional status. The predictive role of this indicator on the prognosis of malignant tumors has only been proposed recently [18,19]. In our study, we cannot get a definitive conclusion whether there is a correlation between COR-BMI and PTC prognosis. This may be related to the disease state of PTC different from other malignant tumors. PTC is a relatively indolent malignancy [7,13]. No previous studies have reported the relationship between RDW and prognosis of PTC. There are not many researches on the inflammation status of PTC. Kari et al. proposed that autoimmune disease, such as Hashimoto’s thyroiditis, may be related to PTC oncogenesis [23]. There is also literature suggesting the role of macrophages and mast cell in the prognosis of PTC tumors [24]. In addition, we found no significant difference in BMI changes between the two prognosis groups during follow-up. This is also consistent with our previous understanding that PTC, unlike other malignant tumors, usually does not experience weight loss (cachexia). In contrast, many studies suggested that obesity is a risk factor for PTC [40,41]. Our study provided new evidence for the insignificant changes in BMI in PTC patients. Previous studies have shown that NLR is associated with poor prognosis of PTC [22]. However, we did not find a significant association between them. There are previous studies that are consistent with our views [42,43]. Increased NLR is associated with inflammatory disease, whereas in non-inflammatory diseases, NLR is within normal range [43]. NLR in our study is within the low and narrow range (median 2.11, IQR 1.56–2.31). This may be the cause of NLR negative results. Further prospective studies will be needed to be performed to validate these conflicting results.

Unexpectedly, we found a significant effect of lower PLT on poor prognosis. Although PLT displayed a detrimental effect on the risk of PPG (OR = 0.996, 95% CI = 0.993–0.999, *P*<0.05). We cannot say that this statistically significant result is clinically significant because both OR and 95% CI are too close to 1. We further divided the PLT values into two groups, of which 302 × 10^9^/l was the cut-off value. After that, PLT subgroups showed a detrimental effect on the risk of PPG (OR = 0.438, 95% CI = 0.264–0.728, *P*<0.05). This statistical difference still existed after we added other covariates that may affect prognosis (*P*<0.05). Therefore, we can conclude that the lower PLT group of patients with PTC has a worse prognosis than the higher PLT group. This result is different from the mainstream view of other solid tumors [20,21], including lung cancer, renal cell carcinoma, gallbladder cancer, rectal cancer, gastric cancer, esophageal squamous cell carcinoma, and ATC.

There are several possible explanations for the pro-tumor effect of PLT in malignancies [20,44]. First, tumor cells can directly induce the activation and aggregation of PLT, promote tumor cell–platelet thrombosis, thereby preventing the immune system from detecting and preventing natural killer cells from clearing intravascular tumor cells, thereby promoting metastasis. This seems to be the main mechanism by which platelets protect tumor cells. Second, thrombin-activated platelets release a variety of growth factors to promote angiogenesis and tumor cell proliferation [20,44]. However, as we discussed earlier, PTC is less invasive and different from other malignant tumors [7,13]. We found the opposite relationship in PTC. We believe this may be related to the association of PLT with radiation sensitivity. Most PTC patients receive ablation of tumor remnants by RAI therapy after thyroidectomy. RAI is not only a key means to remove residual thyroid and metastatic foci, but also an important means to reduce tumor recurrence in patients [5,7]. Therefore, the radiation sensitivity of tumor cells to RAI is important for prognosis. In the course of treatment, some PTC tumor cells will dedifferentiate and become resistant to RAI, and finally become radioiodine-refractory thyroid cancer [2,45]. Resistance to RAI therapy is a central feature of disease recurrence and poor outcomes in thyroid cancer. Studies have shown that platelet-derived growth factor (PDGF), which is mainly synthesized, stored, and released through platelets [46], has a special role in radiation sensitivity of tumors [47,48]. However, the role of PDGF in radiation sensitivity of tumor cells is controversial. On one hand, Studies have shown that increasing the PDGF-receptor can increase the radiation sensitivity of NPC [48]. On the other hand, inhibition of PDGF suppresses tumor growth and enhances tumor radiation response in fibrosarcoma and prostate cancer [47]. Furthermore, McMullen et al. demonstrated that PDGFR-α can induce thyroid follicular cell dedifferentiation [52].Most studies support the view that PDGF promotes resistance to radiation therapy. However, our findings may suggest the possibility of another potential mechanism. Further and more complete research on the relationship between PLT/PDGF and radiation sensitivity is urgently needed.

We noticed that gender did not affect the patient’s prognosis in our results, which is different from previous works’ mainstream viewpoints [49,50]. We thought that the reasons for this difference may be as follows: (1) The deviation of patient selection caused by insufficient sample size. (2) Different hospitals have different references for choosing the dose of RAI. Our department may intentionally impose a higher dose on male patients when giving RAI treatment. In our study, the dose of RAI received by patients was not included in the statistical analysis. We also found that there are also some studies showed that gender is not an independent prognostic factor for survival in PTC [9]. To validate this controversy, we need further research.

Although we have gained some new discoveries, our research still has some limitations. First limitation is retrospective nature and single-center of our study. Second, our study only divided the patient’s prognosis into two groups according to the treatment response, and did not include the patient’s survival time in the study. Further studies with patients’ survival time will access more information about the prognosis. However, PTC patients typically have longer survival times. Survival analysis is difficult to carry out and standardized follow-up will be a big problem. Third, some covariates that influence prognosis should also be included in the study, such as family history, surgical method/scope of surgery [51], the time of the first RAI after surgery, RAI dose, gene mutations etc. Obviously, information on the quality and extent of neck dissection is notably not available. The molecular markers such as BRAF point mutation and TERT promoter point mutation should also be included. Fourth, dynamics of post-treatment changes in these biomarkers should be considered. The prognosis of PTC patients is closely related to the treatment response. These clinical indicators are constantly changing during the course of treatment. We need to further understand the changes of these prognostic indicators. In view of the above limitations, larger multicenter and preferably prospective studies are warranted to validate our findings in the future.

In conclusion, we studied the predictive role of some indicators in the prognosis of patients with PTC. We did not find that COR-BMI and NLR are significant predictors of PTC prognosis. No significant difference in BMI changes was found between the two prognosis groups during follow-up. We found that lower PLT levels may indicate a poor prognosis for PTC. The relationship between PDGF and radiation sensitivity may be the key to this association. Further prospective studies will need to be performed to validate these preliminary results.

## Data Availability

We will provide the original data for non-commercial needs of the journal without breaching participant confidentiality.

## Abbreviations

ATA: American Thyroid Association
ATC: anaplastic thyroid cancer
BMI: body mass index
BRAF: proto-oncogene B-Raf
CI: confidence interval
COR-BMI: combination of RDW and BMI
GPG: Good Prognosis Group
IQR: interquartile range
LBC: lymphocyte
NEUT: neutrophil
NLR: neutrophil–lymphocyte ratio
NPC: nasopharyngeal carcinoma
OR: odds ratio
PDGF: platelet-derived growth factor
PLT: platelet
PPG: Poor Prognosis Group
PTC: papillary thyroid cancer
RAI: radioactive iodine
RDW: red blood cell distribution width
SD: standard deviation
SPSS: Statistical Package for Social Sciences
TERT: telomerase reverse transcriptase
Tg: thyroglobulin
TSH: thyroid stimulating hormone
